# STIL/AURKA axis promotes cell proliferation by influencing primary cilia formation in bladder cancer

**DOI:** 10.1186/s12967-023-04118-2

**Published:** 2023-04-26

**Authors:** Jingxian Li, Yuanjiong Qi, Bo Li, Yan Liu, Kuo Yang, Zhihong Zhang, Jianqiang Zhu, E. Du

**Affiliations:** Tianjin Institute of Urology, The Second Hospital of Tianjin Medical University, Tianjin, China

**Keywords:** PC, Bladder cancer, STIL, AURKA, SHH signaling

## Abstract

**Background:**

The primary cilia (PC) is a microtubule-based and nonmotile organelle which protrudes from the surface of almost all mammalian cells. At present, PC has been found to be a deficiency or loss in multiple cancers. Restoring PC could be a novel targeting therapy strategy. Our research showed that PC was reduced in human bladder cancer (BLCA) cells, and PC deficiency promotes cell proliferation. However, the concrete mechanisms remain unknown. SCL/TAL1 interrupting locus (STIL), a PC-related protein, was screened in our previous study and could influence the cell cycle by regulating PC in tumor cells. In this study, we aimed to elucidate the function of STIL for PC to explore the underlying mechanism of PC in BLCA.

**Methods:**

Public database analysis, western blot, and enzyme-linked immunosorbent assay (ELISA) were used to screen genes and explore gene expression alteration. Immunofluorescence and western blot were utilized to investigate PC. Wound healing assay, clone formation assay, and CCK-8 assay were used to explore cell migration, growth, and proliferation. The co-immunoprecipitation and western blot were employed to reveal the interaction of STIL and AURKA.

**Results:**

We found that high STIL expression is correlated with poor outcomes of BLCA patients. Further analysis revealed that STIL overexpression could inhibit PC formation, activate SHH signaling pathways, and promote cell proliferation. In contrast, STIL-knockdown could promote PC formation, inactivate SHH signaling, and inhibit cell proliferation. Furthermore, we found that the regulatory functions of STIL for PC depend on AURKA. STIL could influence proteasome activity and maintain AURKA stabilization. AURKA-knockdown could reverse PC deficiency caused by STIL overexpression for PC in BLCA cells. We observed that co-knockdown in STIL and AURKA significantly enhanced PC assembly.

**Conclusion:**

In summary, our result provides a potential therapy target for BLCA based on the restoration of PC.

**Supplementary Information:**

The online version contains supplementary material available at 10.1186/s12967-023-04118-2.

## Background

The primary cilia (PC) is a microtubule-based and nonmotile organelle functioning as a cellular antenna that regulates cell proliferation, differentiation, and migration [1–3]. Studies have found that PC protein mutation or dysfunction affects PC formation and is associated with various human diseases. These diseases are collectively called ciliopathies [4]. In multiple human cancers, PC has been found deficient or lost, including pancreatic cancer [5], breast cancer [6], prostate cancer [7], bladder cancer (BLCA) [8], and so on. Our research also observed that PC was deficient in bladder cancer cells compared to bladder normal epithelial cells, and PC deficiency promotes BLCA cell proliferation. However, the mechanisms of PC deficiency in bladder cancer remain unknown.

The beginning of the PC formation is the transformation of the mother centrosome into the basal body. During assembly of the PC, numbers of basal body proteins or centriolar proteins were changed [9]. Many of these centrosome proteins have been shown to have some relationships with cancer [10–12]. PC-related protein—SCL/TAL1 interrupting locus (STIL) is a differential expression gene that we screened from bladder cancer and adjacent normal tissues [8]. STIL is an essential component of the centriole replication machinery and PC formation [13, 14]. STIL (−/−) mouse embryos do not contain centrioles or PC [15]. In our previous study, STIL is up-regulated in tumor tissues and is closely related to the bad prognosis of patients. STIL could regulate PC and affect the cell cycle of kidney and prostate cancer cells [16]. This study found that STIL expression was up-regulated in bladder cancer tissues and cell lines. High STIL expression was correlated with poor outcomes in BLCA patients. STIL knockdown promotes PC formation in BLCA cells and inhibits the PC-mediated SHH pathway. STIL overexpression showed contrary results. These findings indicated that STIL up-regulation might be the reason for PC deficiency in bladder cancer.

Through the gene ontology (GO) enrichment analysis and high-throughput sequencing, we speculated that AURKA might be the downstream proteins of STIL. Aurora kinase A (AURKA), a PC-related gene, was also screened in our previous study [8]. AURKA belongs to the family of serine/threonine kinases, whose activation is necessary for cell division processes via the regulation of mitosis [12]. In bladder cancer, we found that the expression of AURKA is up-regulated. AURKA showed a highly positive correlation with STIL in mRNA expression in BLCA. AURKA expression was affected by STIL expression alteration. In previous research, AURKA was reported as a liable protein and readily degraded by proteasome degradation [17]. We found STIL depletion could enhance proteasome activity and promote AURKA degradation. STIL overexpression showed inhibition of AURKA degradation. These findings indicated that STIL upregulation could maintain AURKA stabilization. Through knocking down AURKA, we observed that deficient PC caused by STIL overexpression was restored, suggesting that STIL could influence PC formation through regulating AURKA. Recent research hypothesizes that restoring PC may be a promising therapeutic strategy in cancers [18]. We found that co-silencing of STIL and AURKA maximizes the restoration of PC in BLCA cells, suggesting these two genes have a synergistic effect on PC regulation. In summary, our research explores the potential function of PC in BLCA cells and provides a novel therapeutic approach for BLCA based on restoring PC.

## Results

### PC are reduced in human bladder cancer cells, and PC assembly of BLCA cells is enhanced under serum-starved conditions

Our previous study found that PC was reduced in BLCA tissues compared with normal tissues [8]. To verify the result, we further examined PC in BLCA cells. By incubating cells with a serum-free medium, the PC assembly of cells was induced. We then used acetylated tubulin as a PC marker in BLCA cell lines (BIU87, EJ, T24, and 5637) and normal bladder epithelial cells (SV-HUC-1). The percentage of ciliated cells was respectively only 13.58% (BIU87), 7.665% (EJ), 9.618% (T24) and 8.356% (5637), while in SV-HUC-1 cells, the percentage of the ciliated cell was 16.85% (Additional file 1: Fig. S1A, B). To investigate whether the PC formation is enhanced in serum-starved BLCA cells, we detected the percentage of ciliated cells in serum-starved EJ cells. We found that the percentage of ciliated cells increased after 48 h serum-starved condition and was restored after serum re-stimulation (Additional file 1: Fig. S1C, D). Moreover, the PC-relate markers (including Acethylated-tubulin and ARL13B) were also up-regulated after 48 h serum-starved condition and was restored after serum re-stimulation (Additional file 1: Fig. S1E).

### PC loss promotes cell cycle and proliferation of BLCA cells

It was reported that IFT88 serves a pivotal role in PC assembly [19, 20]. Silencing IFT88 could construct a function loss model of PC. To elucidate the biological function of PC, we silenced IFT88 in human bladder epithelial immortalized cells (SV-HUC-1) and EJ cells, respectively. We found that the percentage of ciliated cells and protein levels of acetylated tubulin were decreased in IFT88 knockdown cells (Additional file 1: Fig. S2A–D). A previous study has shown that PC is closely related to the cell cycle [16, 21]. IFT88 also has been demonstrated to play a crucial role in regulating the cell cycle [22, 23]. We observed a decrease in the accumulation of the cells in the G0/G1 and an increase in the accumulation of the cells in the G2/M and S phase in IFT88-silencing EJ/T24 cells (Additional file 1: Fig. S2E). Furthermore, IFT88 knockdown significantly promotes BLCA cell proliferation (Additional file 1: Fig. S2F). SHH signaling was a vital pathway that was regulated by PC. Uncontrolled activation of the SHH pathway has been found as a potential oncogenic driver signal promoting the proliferation of cancers [24]. In BLCA cells, we found IFT88 knockdown increases the protein levels of GLI1 and SHH and decreases the protein levels of SMO, revealing that PC deletion induces activation of SHH signaling pathways (Additional file 1: Fig. S2G, H). These results indicated that the decrease of the PC arrested cell cycle, activated the SHH pathway, and promoted proliferation in BLCA cells.

### STIL is significantly upregulated in BLCA and correlated with a poor outcome

**Fig. 1:**
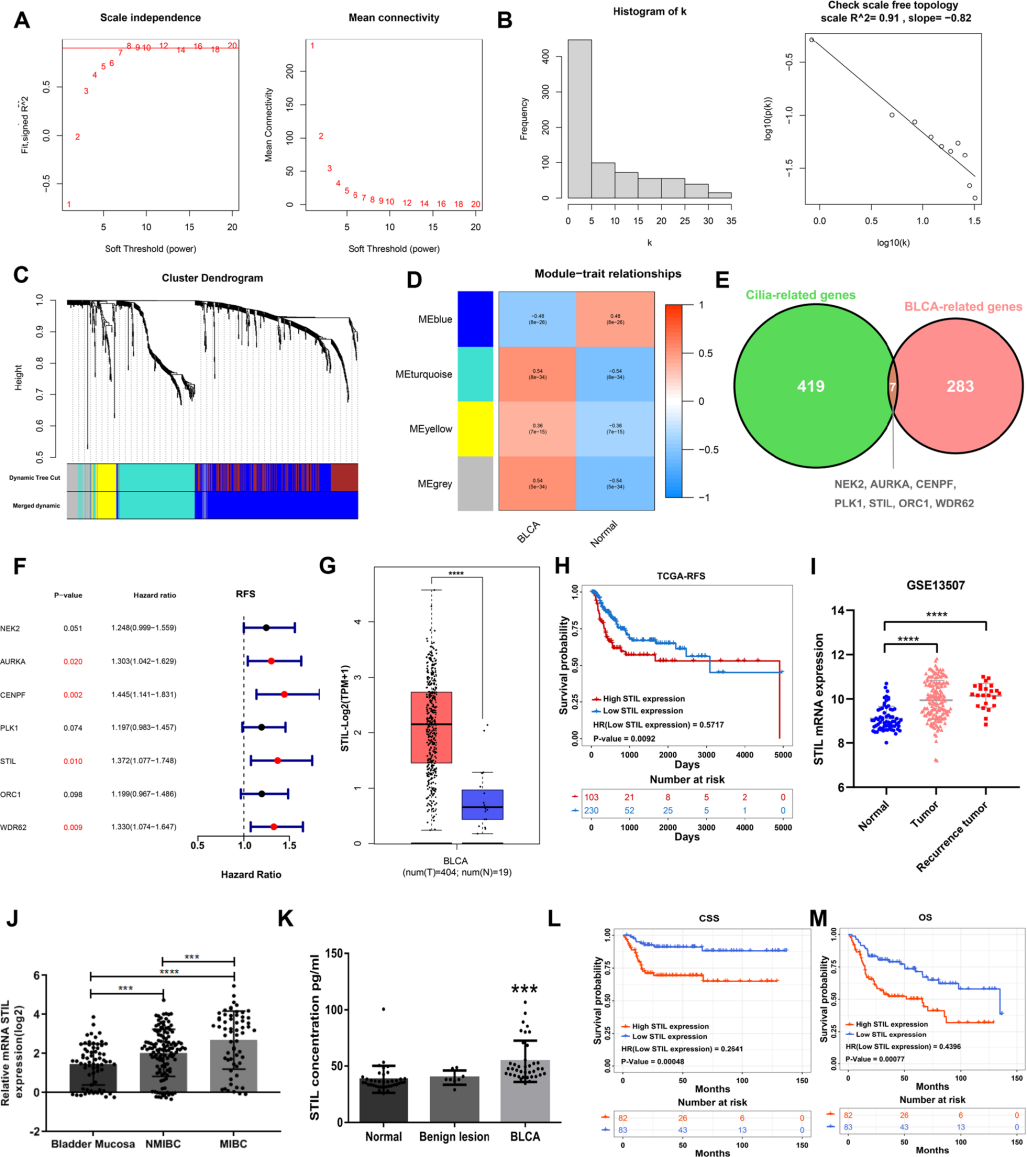
STIL is a potentially essential PC-related protein in BLCA. **A** Analysis of the scale-free fit index and mean connectivity for various soft-thresholding powers in TCGA-BLCA datasets. **B** Checking the scale-free topology when β = 8. K represents the logarithm in the whole network connectivity, and p(k) shows the logarithm of the corresponding frequency distribution. The K value is negatively correlated with p(k). The correlation coefficient r2 was 0.91 in TCGA-BLCA datasets, representing scale-free topology. **C** The Dendrogram of all DEGs in TCGA-BLCA datasets clustered based on a dissimilarity measure (1-TOM). **D** The heatmaps were portrayed to represent the correlation between module eigengenes and clinical status (BLCA and normal status), and p-values and correlation coefficients were shown in every module. **E** Venn diagrams showed 7 overlapping genes between BLCA-related genes and cilia-related genes. **F** The Forest plot represents the hazard ratios of prognosis-related ciliary genes in TCGA-BLCA. The red point indicates the high hazard ratio. P-value < 0.05 is regarded as significant and identified by the red font. **G** Box plot showed the STIL mRNA expression in TCGA-BLCA and normal tissues. **H** The STIL expression data from TCGA-BLCA was divided into high/low expression groups based on the optimal cut-off value by x-tile software. The Kaplan–Meier survival curves were depicted to show disease-free survival (RFS) in high/low STIL expression groups. **I** STIL mRNA expression in normal bladder tissue, bladder cancer, and recurrence bladder cancer tissues from the GSE13507 dataset. **J** STIL mRNA expression in normal bladder mucosa, non-muscular infiltrating bladder cancer, and muscular infiltrating bladder cancer tissues from oncomine database. **K** Detection of the serum STIL factor concentrations in the normal bladder population (Normal group, n = 40), patients with benign bladder disease (NC group, n = 10), and bladder cancer patients (BLCA group) (n = 40). **L**, **M** The STIL expression data from GSE13507 was divided into high/low expression groups based on the median value. The Kaplan–Meier survival curves were depicted to show the cancer-specific survival (CCS) and overall survival (OS) in high/low STIL expression. Unpaired t-test analysis was utilized to compare the differences between the two groups. *P-value < 0.05, **P-value < 0.01, ***P-value < 0.001, ****P-value < 0.0001

To determine the potential PC-related genes in BLCA, we first identified 783 DEGs from the TCGA-BLCA dataset. Then we performed the Weighted Gene Co-expression Network Analysis (WGCNA). To achieve a scale-free co-expression network, we chose the power of β = 8 in TCGA-BLCA datasets (Fig. 1A). We constructed a scale-free topology network when the correlation between k and p(k) reached or exceeded 0.80 (Fig. 1B). Then we merged the highly familiar modules by selecting a cut line of < 0.25 and a minimum module size of 30, utilizing the dynamic hybrid tree cut method (Fig. 1C). We found two modules were highly positively correlated with the BLCA (Turquoise module: r = 0.54, P-value = 8e−34; Grey module: r = 0.54, P-value = 5e−34) (Fig. 1D). We ultimately determined 426 genes in these two modules, 7 of them were PC-related genes (including NEK2, AURKA, CENPF, PLK1, STIL, ORC1, and WDR62) (Fig. 1E). Among these, AURKA, CENPF, STIL, and WDR62 were correlated with the prognosis of BLCA patients (Fig. 1F), we called them prognosis-related PC genes (PRPGs). We first focus on STIL, which has been found to influence cell cycle progression by regulating the PC in kidney and prostate cancer in our previous study [16]. In the TCGA-BLCA dataset, STIL mRNA expression was up-regulated in tumour tissue, and the patients with high STIL expression showed a worse prognosis (Fig. 1G, H). In the other two datasets from the GEO and oncomine database, the STIL mRNA expression was also up-regulated in the tumour, especially the muscle-invasive bladder cancer (MIBC) and recurrence bladder tumour (Fig. 1I, J). We further examined the STIL concentrations in serum of patients with healthy bladder (n = 40, Normal group), patients with benign bladder disease (n = 10, NC group), and BLCA patients (n = 40, BLCA group). As shown in Fig. 1K, STIL levels showed consistent results in the Normal group and NC group. However, STIL concentration represented a significant upregulation in the BLCA group than that in the other two groups (P < 0.05). In the GSE13507 dataset, we also observed that the overall survival (OS), cancer-specific survival (CSS), progression-free survival (PFS), and recurrence-free survival (RFS) of patients with higher STIL expression showed poor outcomes than that of patients with lower STIL expression (Fig. 1L, M, Additional file 1: Fig. S3A, B). Moreover, the high STIL expression group was accompanied by advanced pathologic T stage, pathologic N stage, pathologic M stage, and histology grade (Table 1). Abnormally high STIL expression is associated with increased metastatic potential in multiple cancers [25]. These results imply that high STIL expression plays a tumour-promoting role in BLCA.

**Table 1:** Correlation between STIL mRNA level and clinical characteristics in BLCA patients

	STIL expression				
	High expression	Low expression	Total	χ^2^	P-value
Gender					
Female	22 (26.8%)	8 (9.6%)	30 (18.2%)	7.0794	0.007798
Male	60 (73.2%)	75 (90.4%)	135 (81.8%)		
Age					
< 60	17 (20.7%)	29 (34.9%)	46 (27.9%)	3.4648	0.06269
≥ 60	65 (79.3%)	54 (65.1%)	119 (72.1%)		
Pathologic T					
Ta	5 (6.1%)	19 (22.9%)	24 (14.5%)	18.409	0.001026
T1	36 (43.9%)	44 (53.0%)	80 (48.5%)		
T2	18 (22.0%)	13 (15.7%)	31 (18.8%)		
T3	15 (18.3%)	4 (4.8%)	19 (11.5%)		
T4	8 (9.8%)	3 (3.6%)	11 (6.7%)		
Pathologic N					
N0	69 (84.1%)	80 (96.4%)	149 (90.3%)	5.7278	0.0167
N1-3/Nx	13 (15.9%)	3 (3.6%)	16 (9.7%)		
Pathologic M					
M0	78 (95.1%)	80 (96.4%)	158 (95.8%)	0.00027	0.9869
M1	4 (4.9%)	3 (3.6%)	7 (4.2%)		
Grade					
High grade	48 (58.5%)	12 (14.5%)	60 (36.4%)	32.755	1.046e-08
Low grade	34 (41.5%)	71 (85.5%)	105 (63.6%)		
Invasiveness					
No	40 (48.8%)	63 (75.9%)	103 (62.4%)	11.806	0.0005903
Yes	42 (51.2%)	20 (24.1%)	62 (37.6%)		

### STIL silencing influence proliferation and cell cycle of BLCA cells

**Fig. 2:**
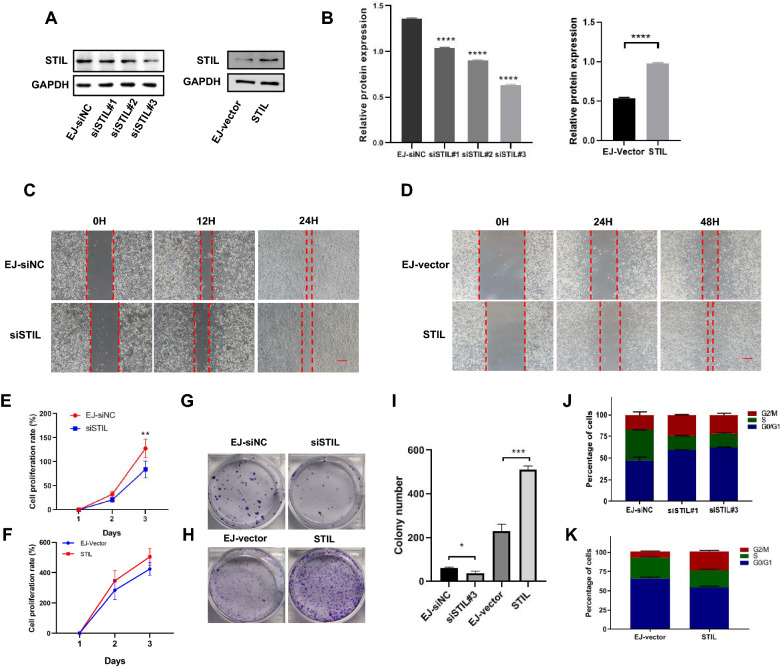
STIL influences the proliferation, migration, cycle, and growth of BLCA cells. **A**, **B** The western blot analysis was performed to validate the STIL expression in STIL knockdown/overexpression compared to their corresponding control, respectively. The percentage of protein levels was quantification. **C**, **D** The migration ability for STIL knockdown/over-expression EJ cells compared to their corresponding control by the wound healing assays. **E**, **F** The viability of STIL knockdown/over-expression EJ cells compared to their corresponding control group was measured by CCK8 assay. **G**–**I** The cell growth for STIL knockdown/over-expression EJ cells compared to their corresponding control group by colony formation assays. The number of colonies was counted utilizing the software image J. **J**, **K** Cell cycle analysis of STIL knockdown/over-expression EJ cells compared to their corresponding control group. Unpaired t-test analysis was utilized to compare the differences between the two groups. *P-value < 0.05, **P-value < 0.01, ***P-value < 0.001, ****P-value < 0.0001

To assess the effect of STIL on the behaviours of bladder cancer cells, three interfering siRNAs and overexpression plasmids were synthesized and evaluated the transfected efficiency by western blot (Fig. 2A, B). After transfecting for 12 h, 24 h, or 48 h, the wound-healing capacity of STIL knockdown BLCA cells significantly decreased compared to the negative control (Fig. 2C and Additional file 1: Fig. S3C). On the contrary, the wound-healing capacity of STIL overexpression cells significant increase compared to the control cells (Fig. 2D and Additional file 1: Fig. S3D). Furthermore, the STIL knockdown in BLCA cells also dramatically decreased cell proliferation and growth. Whereas STIL overexpression obviously increased the proliferation and growth of EJ/T24 cells (Fig. 2E–I; Additional file 1: Fig. S3E–I). STIL is a centriole protein involved in regulating the cell cycle and duplication of centrioles in dividing cells [16, 26], so we further detected the effect of STIL in the cell cycle of BLCA cells. The cell cycle analysis showed that the percentage of cells in the quiescent phase (G0/G1) was higher in STIL-depleted cells and lower in STIL-overexpressed cells compared to normal control, suggesting that STIL is involved in cell cycle G1 to S phase transition in BLCA cells (Fig. 2J, K; Additional file 1: Fig. S3J, K). All in all, silencing STIL restrained the growth of BLCA cells predominantly through inhibition of cell proliferation and induction of cell cycle arrest.

### STIL depletion induces PC assembly, and STIL over-expression induces PC disassembly

**Fig. 3:**
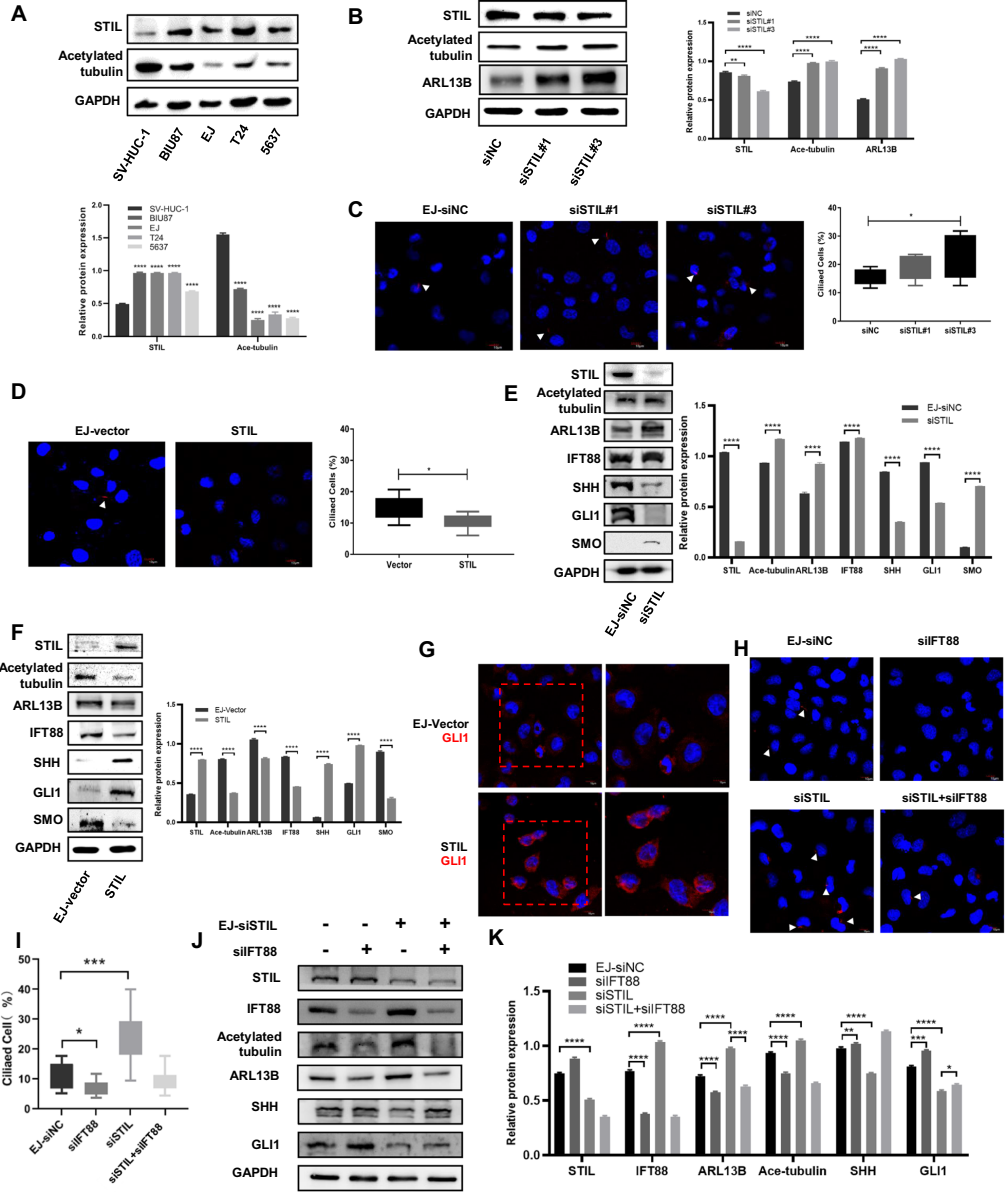
STIL regulates PC formation and the SHH pathway. **A** Western blot analysis for the protein expression (STIL and acetylated tubulin) in SV-HUC-1, BIU87, EJ, T24, and 5637. The percentage of protein levels was quantification. **B** The protein expression levels of STIL, acetylated tubulin, and ARL13B in STIL-knockdown and control EJ cells. The percentage of protein levels was quantification. **C**, **D** The STIL knockdown/overexpression and corresponding control EJ cells were serum-starved for 48 h, then fixed and stained antibodies against ace-tubulin (red) and DAPI (blue) for immunofluorescence analysis. The percentage of ciliated cells were compared between the control and treatment group. **E** Western blot analysis for the protein expression (including STIL, IFT88, acetylated tubulin, ARL13B, SHH, SMO, and GLI1) in STIL-knockdown and control EJ cells. The percentage of protein levels was quantification. **F** Western blot analysis for the protein expression (including STIL, IFT88, acetylated tubulin, ARL13B, SHH, SMO, and GLI1) in STIL-overexpression and control EJ cells. The percentage of protein levels was quantification. **G** Control and STIL overexpression EJ cells were fixed and stained with antibodies against GLI1 for immunofluorenscence analysis. **H**, **I** siNC and siSTIL EJ cells transfected with siIFT88 or not, respectively, were serum-starved for 48 h, then fixed and stained with antibodies against ace-tubulin (red) and DAPI (blue) for immunofluorescence analysis. The scale bar indicates 10 μm. The percentage of ciliated cells was quantification. **J**, **K** The protein expression levels (including STIL, acetylated tubulin, ARL13B, IFT88, SHH, and GLI1) in siNC and siSTIL EJ cells transfected with siIFT88 or not were respectively detected by western blot analysis. All the percentages of protein levels were quantification. Unpaired t-test analysis was utilized to compare the differences between the two groups. *P-value < 0.05, **P-value < 0.01, ***P-value < 0.001, ****P-value < 0.0001

STIL is a cilia-related protein which is essential for PC formation [14]. To explore the effects of STIL for PC in BLCA, we firstly investigated STIL and acetylated tubulin (a ciliary marker) expression in one bladder epithelial cell (SV-HUC-1) and four BLCA cell lines (BIU87, EJ, T24, and 5637). Compared with SV-HUC-1 cells, STIL expression was up-regulated in BLCA cells (Fig. 3A). In contrast, the expression of acetylated tubulin was lower in BLCA cells. What is more, compared with siNC-transfected cells, STIL depleted cells showed an increased expression of acetylated tubulin and ARL13B (Fig. 3B). To address where STIL localizes exactly, STIL location was compared with the location of markers including γ-tubulin (centriole) and acetylated tubulin (PC). The results showed that STIL localized at centriole of BLCA cells (Additional file 1: Fig. S4A), which is consistent with the previous study [13]. As shown in Additional file 1: Fig. S1E, PC assembly could be induced by serum starvation in BLCA cells. To investigate whether STIL expression is regulated by those conditions, STIL expression was tested in different serum starvation times. The results showed that STIL expression was down-regulated after 24 h under serum-starved conditions and restored after serum re-stimulation (Additional file 1: Fig. S4B). Immunofluorescent staining indicated STIL expression decreased in the quiescent phase (G0/G1) and was more in the mitotic phase (G2/M) (Additional file 1: Fig. S4C). So a contrary appearance has existed between STIL expression and PC formation in the BLCA cell cycle. We further investigated the alterations of PC in STIL depletion or STIL overexpression of BLCA cells. Under 48 h serum-starved conditions, the percentage of ciliated cells was increased in STIL depletion BLCA cells (Fig. 3C; Additional file 1: Fig. S4D). In comparison, the percentage of ciliated cells was decreased in STIL overexpression BLCA cells (Fig. 3D; Additional file 1: Fig. S4E). GLI1 is a downstream effector of SHH signalling pathway, we found that STIL silencing inhibited GLI1 expression, and STIL upregulation activated GLI1 expression, indicating that SHH signalling pathway was regulated by STIL expression alteration in BLCA cells (Fig. 3E–G; Additional file 1: Fig. S4F, H). Furthermore, we observed that the effect of STIL silencing on PC and SHH signalling could be reversed by silencing IFT88 (Fig. 3H–K; Additional file 1: Fig. S4I–K), revealing the regulatory function of STIL on PC is dependent on IFT88.

### STIL depletion induces the expression of PC-related proteins and inhibit tumor growth in vivo

**Fig. 4:**
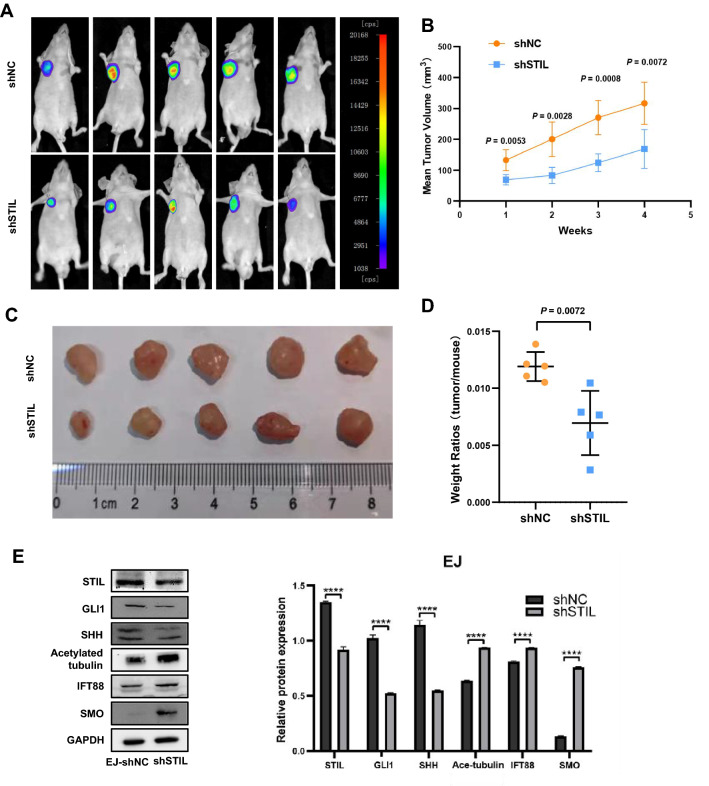
STIL depletion inhibits tumour growth in vivo. **A** Bioluminescent images of xenograft bladder-tumor mouse models implanted with STIL depletion cells. **B** The volume of bladder tumour in 1–4 weeks. **C**, **D** Photograph of xenograft subcutaneous bladder tumours (left). Statistical analysis of the tumour weights (right). **E** The western blot analysis of xenograft subcutaneous bladder tumours' protein expression (STIL, GLI1, SHH, Acetylated tubulin, IFT88, and SMO). The percentage of protein levels was quantification. Unpaired t-test analysis was utilized to compare the differences between the two groups. *P-value < 0.05, **P-value < 0.01, ***P-value < 0.001, ****P-value < 0.0001

To observe the tumorigenicity of STIL in vivo, we established the xenograft models by injecting stable shSTIL EJ cells and shNC EJ cells into subcutaneous tissues of nude mice. No significant body loss was observed during the treatment in each group. Tumour volume measurements were used to monitor the tumour progression. After 4 weeks, the bioluminescence imaging (BLI) showed that all nude mice developed xenogeneic tumours at the injection site (Fig. 4A). As shown in Fig. 4B, STIL down-regulation decreased the xenograft tumour volume compared to the control group. Furthermore, Tumour growth and weight ratios (weight of tumour/weight of mouse) of the STIL silenced group showed slower than that of the vector-transfected group (Fig. 4C, D). Western blotting showed the restoration of acetylated tubulin and downregulated HH signalling pathways caused by STIL depletion (Fig. 4E). These results indicated that STIL depletion plays a pivotal role in inhibiting tumorigenicity of bladder cancer in vivo.

### STIL induced cilia disassembly through activating AURKA in BLCA cells

**Fig. 5:**
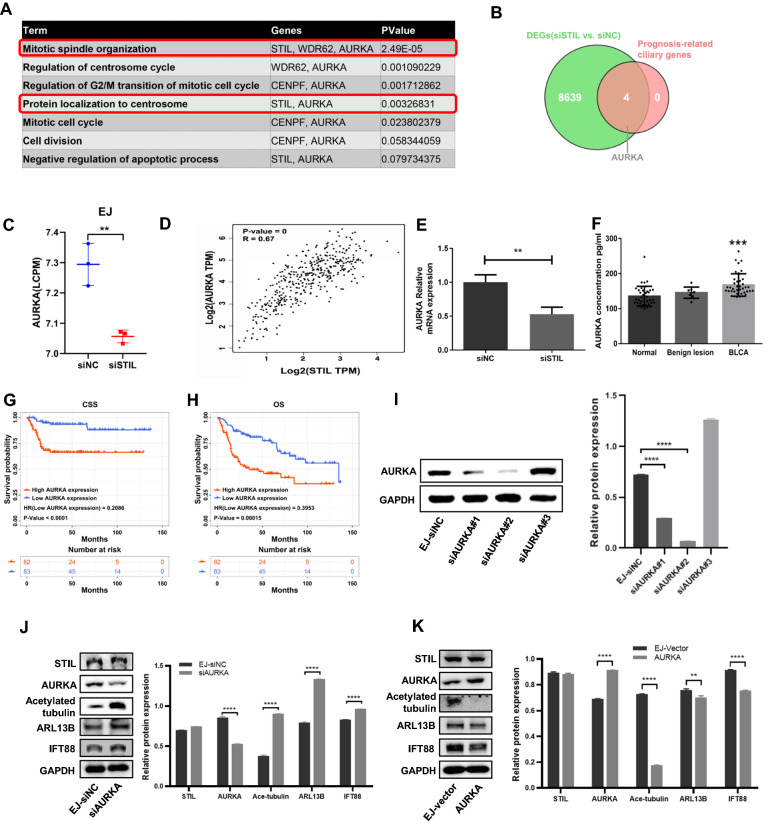
AURKA is up-regulated in BLCA tissue and is highly correlated with STIL. **A** Biological progression enrichment analysis of four prognosis-related ciliary genes (STIL, AURKA, WDR62, and CENPF). **B** Venn diagram reveals the overlapping genes between DEGs of RNA-seq data and prognosis-related ciliary genes. **C** AURKA mRNA expression was downregulated in STIL knockdown EJ cells based on the RNA-seq analysis. **D** The Pearson correlation coefficient (PCC) showed the co-expression relationship between STIL and AURKA mRNA expression in the TCGA-BLCA datasets. **E** qRT-PCR assay reveals that AURKA was down-regulated by silencing STIL in bladder cancer cell. **F** Detection of the serum AURKA factor concentrations in the normal bladder population (Normal group, n = 40), patients with benign bladder disease (NC group, n = 10), and bladder cancer patients (BLCA group) (n = 40). **G**, **H** The AURKA expression data from GSE13507 was divided into high/low expression groups based on the median value. The Kaplan–Meier survival curves were depicted to show the cancer-specific survival (CCS) and overall survival (OS) in high/low AURKA expression. **I** The expression of AURKA protein in transfected and control cells was detected by western blot after transfection of EJ cells with lipofectamine 8000 reagent. **J**, **K** Western blot analysis for the protein expression (STIL, AURKA, IFT88, acetylated tubulin, ARL13B) in the AURKA-knockdown/AURKA overexpression groups. The percentage of protein levels was quantification. Unpaired t-test analysis was utilized to compare the differences between the two groups. *P-value < 0.05, **P-value < 0.01, ***P-value < 0.001, ****P-value < 0.0001

To elucidate the underlying molecular mechanism of STIL for regulating PC in BLCA cells, we conducted the GO enrichment analysis using the four PRPGs mentioned above (Fig. 5A). We finally concentrated on the PC-related protein, AURKA. The same as STIL, AURKA participate in the biological progression of mitotic spindle organization and protein localization to the centrosome. We speculated that STIL and AURKA play the same roles on PC. We further conducted RNA-seq to study the role of STIL in BLCA cells and identified 8639 DEGs in EJ cells after STIL knockdown. Among these, we found the AURKA was down-regulated under STIL-knockdown circumstance (Fig. 5B, C). Furthermore, the mRNA expression of AURKA was highly correlated with STIL in TCGA-BLCA datasets (PCC = 0.67, P-value = 0) (Fig. 5D), silencing STIL could decrease the mRNA expression levels of AURKA (Fig. 5E). These results revealing that AURKA may be downstream of STIL. We further detected the AURKA concentrations in patients with healthy bladder (n = 40; Normal control), benign bladder disease (n = 10; NC group), and bladder cancer (n = 40; BLCA group). The concentrations of AURKA in the BLCA group were higher than that in the Normal control and NC group (P < 0.01) (Fig. 5F). In GSE13507 datasets, the patients with high AURKA expression showed a worse prognosis (including OS, CSS, PFS, and RFS) than those with low AURKA expression (Fig. 5G, H; Additional file 1: Fig. S5A, B). Furthermore, the gender, pathologic T, pathologic M, pathologic N, histologic grade, and invasiveness showed different percentages in high/low AURKA expression. The high AURKA expression group showed a high advanced pathologic stage and histologic grade (Table 2). In STIL depletion or overexpressed cells, the AURKA protein level was decreased or overexpressed, revealed AURKA expression was regulated by STIL (Additional file 1: Fig. S5C, D). Subsequently, three interfering siRNAs and overexpression plasmids of AURKA were synthesized and evaluated the transfected efficiency by western blot (Fig. 5I). We found that AURKA silencing increases the expression of acetylated tubulin, IFT88, and ARL13B, whereas AURKA overexpression decrease the expression of acetylated tubulin, IFT88, and ARL13B (Fig. 5J, K; Additional file 1: Fig. S5E, F). However, STIL expression was not affected by AURKA expression alteration. These results indicated that AURKA is a downstream effector of STIL in BLCA cells.

**Table 2:** Correlation between AURKA mRNA level and clinical characteristics in BLCA patients

	AURKA expression				
	High expression	Low expression	Total	χ^2^	P value
Gender					
Female	23 (28.0%)	7 (8.4%)	30 (18.2%)	9.3906	0.002181
Male	59 (72.0%)	76 (91.6%)	135 (81.8%)		
Age					
< 60	18 (22.0%)	28 (33.7%)	46 (27.9%)	2.2927	0.13
≥60	64 (78.0%)	55 (66.3%)	119 (72.1%)		
Pathologic T					
Ta	5 (6.1%)	19 (22.9%)	24 (14.5%)	28.901	8.186e-06
T1	33 (40.2%)	47 (56.6%)	80 (48.5%)		
T2	18 (22.0%)	13 (15.7%)	31 (18.8%)		
T3	18 (22.0%)	1 (1.2%)	19 (11.5%)		
T4	8 (9.8%)	3 (3.6%)	11 (6.7%)		
Pathologic N					
N0	68 (82.9%)	81 (97.6%)	149 (90.3%)	8.5232	0.003506
N1-3/Nx	14 (17.1%)	2 (2.4%)	16 (9.7%)		
Pathologic M					
M0	78 (95.1%)	80 (96.4%)	158 (95.8%)	0.00026852	0.9869
M1	4 (4.9%)	3 (3.6%)	7 (4.2%)		
Grade					
High grade	51 (62.2%)	9 (10.8%)	60 (36.4%)	44.812	2.169e-11
Low grade	31 (37.8%)	74 (89.2%)	105 (63.6%)		
Invasiveness					
No	37 (45.1%)	66 (79.5%)	103 (62.4%)	19.364	1.08e-05
Yes	45 (54.9%)	17 (20.5%)	62 (37.6%)		

**Fig. 6:**
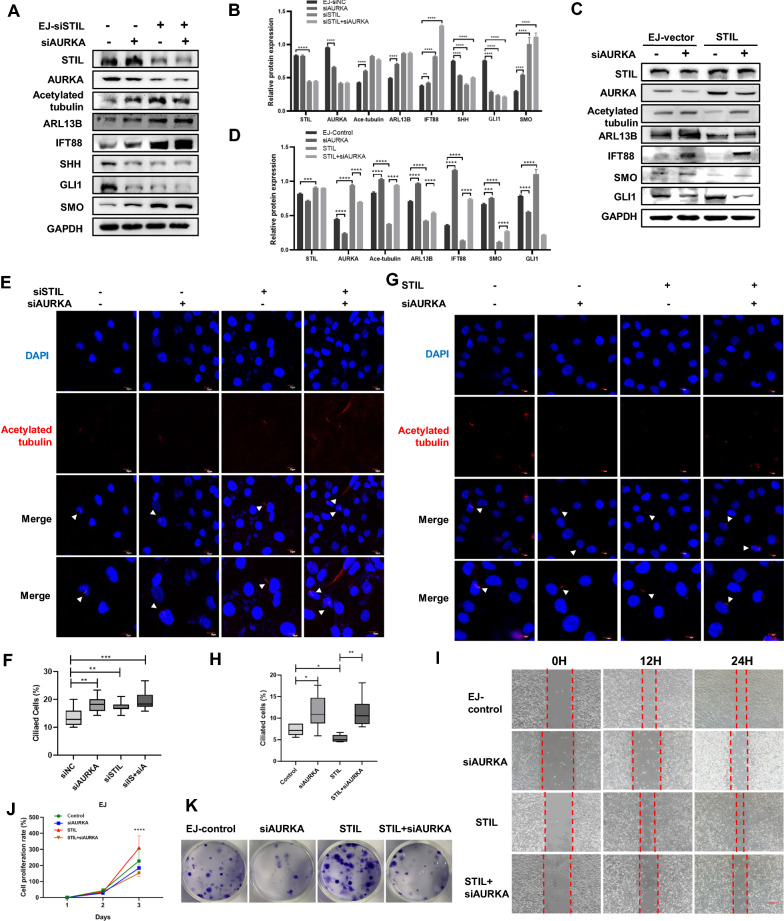
STIL influences PC formation by regulating AURKA. **A**, **B** The protein expression levels (STIL, AURKA, acetylated tubulin, ARL13B, IFT88, SHH, GLI1, and SMO) in siNC and siSTIL EJ cells were transfected with siAURKA or not, respectively, were detected by western blot analysis. **C**, **D** The protein expression levels (STIL, AURKA, acetylated tubulin, ARL13B, IFT88, SHH, GLI1, and SMO) in control and STIL over-expressed EJ cells transfected with siAURKA or not, respectively, were detected by western blot analysis. All the percentages of protein levels were quantification. **E**, **F** siNC and siSTIL EJ cells were transfected with siAURKA or not, respectively, were serum-starved for 48 h, then fixed and stained with antibodies against ace-tubulin (red) and DAPI (blue) for immunofluorescence analysis. The scale bar indicates 10 μm. The percentage of ciliated cells was quantification. **G**, **H** Control and STIL overexpression EJ cells were transfected with siAURKA or not, respectively, were serum-starved for 48 h, then fixed and stained with antibodies against ace-tubulin (red) and DAPI (blue) for immunofluorescence analysis. The scale bar indicates 10 μm. The percentage of ciliated cells was quantification. **I**, **J** Control and STIL over-expressed EJ cells transfected with siNC or siAURKA were analyzed by wound-healing assay. The viability of control and STIL over-expressed EJ cells transfected with siNC or siAURKA, respectively, were analyzed by CCK8 assay. **K** The cell growth of control and STIL over-expressed EJ cells transfected with siNC or siAURKA were analyzed by clone formation assay. Unpaired t-test analysis was utilized to compare the differences between the two groups. *P-value < 0.05, **P-value < 0.01, ***P-value < 0.001, **** P-value < 0.0001

To further confirmed whether STIL inhibits PC formation through AURKA in BLCA cells, we next transfected AURKA siRNA in STIL-depleted or STIL-overexpressed cells (Fig. 6A–D, Additional file 1: Fig. S5G, H). The data showed that STIL expression was not affected by siAURKA. Co-knockdown of AURKA and STIL in BLCA cells results in the significantly increased expression of cilia-associated proteins and decreased expression of the SHH pathway. In contrast, interference with AURKA in STIL-overexpressed cells increased the expression of PC proteins and reversed the activated HH pathway caused by STIL-overexpressed. Furthermore, co-knockdown of these two proteins produced the obviously stimulatory effect on ciliogenesis in BLCA cells (Fig. 6E, F; Additional file 1: Fig. S6A, B). Moreover, AURKA depletion could rescue the cilia reduction, cell proliferation, growth, and migration affected by STIL overexpression (Fig. 6G–K, Additional file 1: Fig. S6C–G). Collectively, these indicated that STIL could influence PC formation through regulating AURKA in BLCA cells.

### STIL interacts with AURKA and enhances AURKA stability by blocking AURKA degradation

**Fig. 7:**
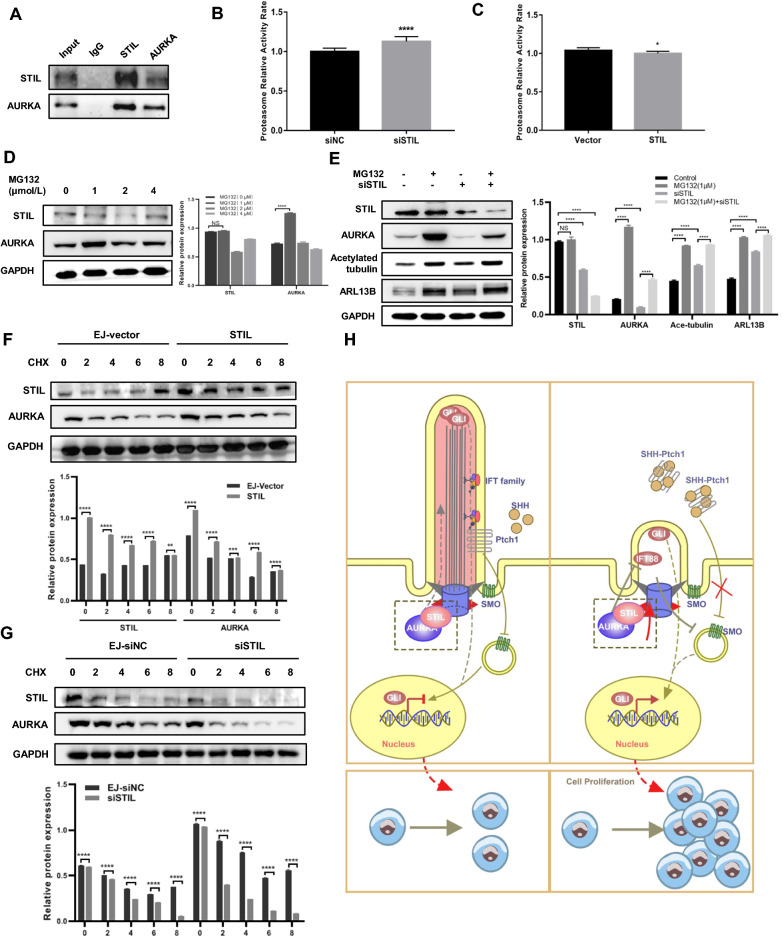
STIL interacted with AURKA and could maintain AURKA stabilization. **A** Co-Immunoprecipitation analysis shows that STIL can interact with AURKA. **B**, **C** Proteasome activity assay of STIL knockdown/overexpression and their corresponding control. **D** The western blot analysis for the protein expression (STIL and AURKA) in EJ cells was treated with different concentrations of MG132 (0, 1, 2, 4 μM). **E** The western blot analysis for the protein expression (STIL, AURKA, acetylated tubulin, and ARL13B) in siNC and siSTIL EJ cells treated with MG132 or not. **F**, **G** The western blot analysis for protein expression (STIL and AURKA) in STIL overexpression/STIL knockdown and their corresponding EJ cells treated with different concentrations of cycloheximide (CHX). All the percentages of protein levels were quantification. Unpaired t-test analysis was utilized to compare the differences between the two groups. **H** Schematic diagram of the STIL/AURKA/PC/SHH signalling in this study. *P-value < 0.05, **P-value < 0.01, ***P-value < 0.001, ****P-value < 0.0001

To delineate the interaction of STIL and AURKA, we performed a co-immunoprecipitation (Co-IP) experiment. After IP of endogenous STIL and AURKA from EJ cells, we detected the presence of endogenous AURKA and STIL, respectively (Fig. 7A). AURKA is a liable protein and readily degraded by proteasome degradation [17]. STIL overexpression increases the protein level of AURKA, so we asked whether STIL blocked AURKA degradation. A proteasome activity assay was firstly performed, and the results revealed increased proteasome activity in STIL depletion EJ cells and decreased proteasome activity in STIL overexpression cells (Fig. 7B, C). We then treated EJ cells with proteasome inhibitor MG132 and found that the up-regulated protein level of AURKA but STIL was not affected (Fig. 7D). Furthermore, in the normal control and STIL depletion EJ cells, respectively treated with MG132 or not, we found that AURKA, acetylated tubulin, and ARL13B expression was restored in control and STIL depletion EJ cells (Fig. 7E). These findings indicated that STIL deficiency promotes AURKA degradation through utilizing proteasome in BLCA cells. After cycloheximide (CHX) treatment to block new protein synthesis, AURKA levels were stabilized in the presence of STIL, however, which were degraded rapidly in cells transfected with a siSTIL (Fig. 7F, G; Additional file 1: Fig. S6H–K). These results suggest that STIL could maintain AURKA stabilization by blocking its degradation. All in all, our research revealed that the STIL-AURKA axis inhibits IFT88 to promote degradation of PC and activates SHH signalling to promote cell proliferation of BLCA (Fig. 7H).

## Discussion

PC is a microtubule-based, non-motile organelle that protrudes as a solitary unit from the basal body in the human body [27]. PC can detect alterations in the extracellular environment, deliver signalling information to the cell, and regulate cell differentiation, developmental, and physiological processes [1–3]. The dysfunction of PC is associated with a wide range of human diseases known as ciliopathies [4]. In recent years, due to the high proliferation phenotype of the tumor, PC deficiency has been detected in various cancers, and researchers have begun to explore the mechanism of PC deficiency [5–7, 16, 21]. However, PC also can be detected in cancer cells [28]. Like the medulloblast and basal cell carcinoma, PC was required for or to inhibit tumor formation, depending on the initiating oncogenic event [28, 29]. Other researchers indicate that PC was reduced in multiple tumors, restoring PC might be a tumor therapeutic strategy [18]. Therefore, PC plays a complex role in promoting and preventing tumorigenesis [28]. Excavating the potential mechanism of PC existence or deficiency in the different tumors could provide a foundation for targeted therapeutic strategy in the future.

BLCA has become common globally due to its prevalence, high recurrence risk, and treatment failures [30, 31]. A previous study found that PC assembly correlates with EMT beginning from urothelial basal cells, indicating a potential implication of PC in BLCA migration and invasiveness [32]. However, in our previous study, though PC presence could be detected, the number of PC in bladder cancer tissues and cells declined compared to normal bladder tissues and epithelial cells. The substantial role of this controversial structure present in bladder cancer still remains unclear. The loss-of-function models of PC could be established by silencing IFT88, a member of IFT subcomplex B subunits [20, 33]. SHH signalling is the most critical pathway regulated by primary cilia [34], which is activated in multiple PC-deficiency cancers such as skin, brain, liver, colon, breast, lung, prostate, and hematological malignancies. The absence of cilia could result in tumorigenesis by expressing an activator form of one of the GLI proteins (a downstream effector of SHH pathways) to activate the SHH signalling [35]. Therefore, the existence of PC is essential for SHH signalling in regulating tumorigenesis. Our results revealed that PC loss caused by IFT88 silencing could promote SHH signalling pathways activated in bladder cancer cells and uncontrolled cell proliferation. These findings provided a possibility to construct a link between PC, bladder cancer, and SHH signalling. However, the influence on abnormal signalling pathway regulation caused by PC loss in tumor formation and development remains unclear. Multiple PC-related proteins regulated PC. Evidence has indicated that some PC-related protein dysfunction could promote cancer development, such as PLK1, PLK4, AURKA, and so on [10–12]. Identifying a new regulator of the PC could provide a better channel to excavate the molecular mechanism underlying PC disassembly in cancers.

The balance of PC-related protein STIL is essential for tumor and signalling pathways. Absolute loss of STIL causes no centrosomes, no cilia, and is not compatible with life [14]. However, STIL was overexpressed in multiple cancers with PC deficiency [16]. We previously found that STIL silencing could restore PC formation, which is a ciliary-dependent mechanism, as cells that were transfected with siRNA targeting the ciliary protein IFT88 interrupted ciliogenesis by STIL silencing, and the PC phenotype and SHH pathways could be reversed [16]. So balancing STIL expression is essential for primary cilia existence and tumor growth. Though silencing STIL expression to control STIL reach to specific levels instead of absolute loss not only promote PC formation but inhibit tumor progression. In bladder cancer, we also found STIL was up-regulated. The patients with high STIL expression showed a worse prognosis. STIL silencing induces PC assembly, prevents G0/G1 phase cells transfer to the G2/M phase, inhibits SHH signalling and causes cell proliferation and growth inhibition in bladder cancer cells. Furthermore, STIL overexpression decreases PC formation and induces G0/G1 phase cells to transfer to the G2/M phase, activate SHH signalling, and promote BLCA cell proliferation. Some studies have revealed that STIL up-regulation presumably affects the formation of mitotic spindles as well as SHH signalling and the function of its interactors [36]. Downregulation of STIL decreases CDK1/CCNB1 activity and prevents G2/M transition [37]. These results indicated the critical role of STIL in regulating PC in bladder cancer. When primary cilia are present, SMO and GLI1 will control SHH signalling. Interestingly, we observed that SMO was upregulated and GLI1 was downregulated under STIL depletion. Moreover, STIL overexpression could inhibit SMO expression and induce GLI1 overexpression. In fact, the primary cilia play a dual role in promoting and preventing tumorigenesis within the domains of the SHH signalling pathway [38]. In basal cell carcinoma, PC loss could inhibit tumor growth induced by the activated form of the SMO upstream activator. However, PC loss could induce an activated form of GLI to cause tumor growth. When medulloblastoma was driven by SMO, PC removal will inhibit tumor formation, whilst PC removal was required for tumor growth when the tumor was driven by GLI [29]. So the tumor-promoting and preventing role of PC depends on the initiating carcinogenic factor. We speculated that PC deficiency due to STIL overexpression could inhibit the activated form of SMO and induce the activated form of GLI1 to promote tumorigenesis in bladder cancer. Of note, we observed that GLI1 overexpression mainly located in cytoplasmic in STIL overexpressed cells, suggesting that SHH signalling activation is correlated with the nuclear translocation of GLI1 [39].

AURKA also is a cilia-related differential expression gene (DEG) in our research, which is co-expressed with STIL. Previous studies have revealed that inhibiting AURKA expression could reverse PC deficiency [40, 41]. In addition, the AURKA inhibitor could rescue ciliogenesis and reverse the acquiring resistance to sonidegib (SMO inhibitor) of tumor cells due to PC deficiency [42], revealing the essential role of AURKA on tumor cell PC formation. AURKA mainly localizes around the centrosome and the microtubule region near the centrosome [41]. The majority of the AURKA population is activated in the late G2 phase. During mitosis, AURKA proteins move along the mitotic spindle [43]. In bladder cancer cells, AURKA expression was affected by STIL. AURKA deletion/overexpression causes the same phenotype, such as PC assembly, cell proliferation and growth, and SHH signalling inhibition/activation. Furthermore, AURKA depletion could reverse the malignancy phenotype, PC disassembly, and SHH signalling activation induced by STIL overexpression in BLCA cells, suggesting that STIL regulation for PC is dependent on AUKRA in bladder cancer.

Our further study found that STIL depletion can promote proteasome activation and AURKA degradation. AURKA protein stabilization has been reported to be associated with the ubiquitin–proteasome system (UPS) [44]. After treatment with cycloheximide (CHX) to block new protein synthesis, AURKA was found decreased degradation in STIL depletion cells and increased degradation in STIL over-expressed cells. These results revealed that STIL promotes AURKA expression by maintaining the stabilization of AURKA in bladder cancer cells.

PC has widely been known as a tumour suppressor organelle [45]. Recent research hypothesizes restoring cilia may be a promising therapeutic strategy in cancers [18]. There are some pre-clinical examples of PC restoration as a therapeutic target, such as PC restoration by HDAC inhibitors in cholangiocarcinoma decrease cholangiocarcinoma cell growth [21, 46]. In HH pathway-dependent cancers, PC loss is a mechanism of resistance to Smo inhibitors [47]. In addition, multiple compounds (gefitinib and dexamethasone) that could restore cilia have been verified in cell lines of multiple human cancers (such as pancreatic, breast, kidney, and lung cancers) [48]. These compounds could inhibit cell proliferation in different cancer cells. However, whether these drugs play a therapeutic role in restoring PC remains to be explored. In our study, STIL or AURKA deletion can cause PC restoration in BLCA cells. In contrast to silencing individual genes, co-silencing in STIL and AURKA produces more encouraging results in bladder cancer cells. Therefore, it is believed that targeting these two genes may be considered an effective strategy for bladder cancer by restoring PC.

## Conclusions

In summary, we found that STIL is highly expressed in BLCA, which could regulate PC formation, and promote cell migration, growth, and cycle. STIL up-regulated inhibits PC formation, activates the downstream factor of the SHH pathway, GLI1, and then promotes cell proliferation. Furthermore, we found that STIL regulation for PC depends on AURKA. AURKA knockdown could reverse the phenotype caused by STIL overexpression. Co-knockdown of STIL and AURKA could maximize the PC restoration in BLCA cells. PC restoration was regarded as a novel therapeutic strategy. Our research provides a potential therapy target for BLCA based on recovering PC.

## Methods

### Data collection

We downloaded the mRNA expression profiles of BLCA from the TCGA (https://xena.ucsc.edu/), GEO (https://www.ncbi.nlm.nih.gov/gds) database, and oncomine database (http://www.oncomine.org/). The TCGA-BLCA expression value was normalized and transformed to a CPM unit by utilizing the edgR packages, and then the data were conducted for subsequent differential expression analysis. The GSE13507 dataset obtained from the GEO and oncomine databases was downloaded for subsequent validation.

### Differential expression analysis and the weighted gene co-expression network analysis

The differential expression genes (DEGs) between TCGA-BLCA and adjacent normal tissues were screened using the limma R-package downloaded from the Bioconductor (https://www.bioconductor.org/). DEGs were filters using the |log2(Fold change)| > 1 and adjusted P-value < 0.05. 783 DEGs were screened and subjected to the WGCNA package to identify the key co-expression modules. The Pearson analysis was conducted to establish a matrix of similarity based on the DEGs. The power value (β = 8) was used to obtain an adjacency matrix (AM) and a Topological overlap matrix (TOM). When the correlation between k and p(k) reached or over 0.80, the ideal power value was determined to build a scale-free topology network. Subsequently, the similar gene expressions were stratified into different gene co-expression modules by the hierarchical clustering dendrogram of the 1-TOM matrix. For any modules, since the module Eigengenes (ME) offered the most appropriate interpretation of the gene expression profile, the ME was correlated with clinical features, which included BLCA or normal status in our study. Finally, we selected the modules displaying highly positive correlation (according to Moduletrait relationships) as further research goals.

### Data analysis

STIL and AURKA mRNA expression data were extracted from the TCGA-BLCA dataset. The Pearson Correlation Coefficient (PCC) was calculated to show the correlation between STIL and AURKA mRNA expression from the TCGA BLCA dataset. The TCGA-BLCA and GSE13507 were utilized to analyze the relationship between clinical information and gene expression. Then the expression of STIL and AURKA were divided into two groups based on the ideal cut-off value determined by x-tile software or the median of their mRNA expression. The distribution of various clinical information in the two groups was analyzed, and a chi-square test was performed to show the significance of the distribution. The univariate Cox analysis was performed to assess the correlation between STIL/AURKA expression and patient survival status (including OS, overall survival; CSS, cancer-specific survival; PFS, progression-free survival; RFS, recurrence-free survival). Moreover, the Kaplan–Meier survival curves were plotted using the R software. A log-rank test was performed to estimate the survival difference by comparing the high STIL/AURKA expression cohort with the low STIL/AURKA expression cohort.

### Cell culture

Human bladder cancer (BLCA) cell lines (BIU87, EJ, T24, and 5637) and normal uroepithelial cell lines SV-HUC-1 were purchased from the Cell Resource Center Affiliated with the Chinese Academy of Medical Sciences. BLCA cell lines were cultured in RPMI-1640 medium (HyClone, Logan, UT, United States), and SV-HUC-1 was cultured in Ham’s F-12 nutrient medium (Ham’s F12K, Sigma‐Aldrich, St. Louis, MO) in a humidified atmosphere incubator of 5% CO_2_ at 37 °C. Both the mediums are supplemented with 10% fetal bovine serum (HyClone, Logan, UT, United States) and 5% penicillin–streptomycin (100 units/ml). The cell culture medium was renewed every 1–2 days depending on the cell density. The cells were divided into a ratio of 1:3–4 when they reached 80–90% gather. All cells were mycoplasma free and authenticated by STR profiling.

### Transfection

STIL, AURKA, and IFT88 were silenced using siRNA oligonucleotides. The target sequences have been listed in Additional file 3: Table S1. The lentivirus vectors of STIL knockdown and overexpression were purchased from GenePharma. STIL knockdown lentivirus was termed shSTIL, and negative control was called shNC. STIL overexpression lentivirus was termed STIL, and negative control was called a vector.

### Protein extraction and western analysis

The cells were gained and lyzed in six-well plates using a total protein extraction reagent with 10% PMSF after transfecting for 48 h. Then the cell suspensions were centrifuged and taken with the supernatant. The total proteins’ supernatant was then used to detect the concentrations by the BCA assay. Extracted proteins were separated by sodium dodecyl sulphate–polyacrylamide gel electrophoresis (SDS-PAGE) on 10% gels and transferred electronically onto polyvinylidene fluoride (PVDF) membranes (Invitrogen). The membranes were incubated with 5% skimmed milk powder diluted by TBST buffer at room temperature for 1 h. Then the membranes were incubated with antibodies against STIL, Acetylated-Tubulin, ARL13B, IFT88, SHH, AURKA, GLI1, SMO, and GAPDH at 4 °C overnight, respectively. The bound antibodies were detected with HRP-conjugated secondary antibodies and visualized using Immobilon™ western chemiluminescent HRP Substrate. Finally, the ECL Western blotting detection system was utilized to visualize the immunoreactive bands. The intensities of the detected bands were calculated using the ImageJ program.

### Wound healing assay

The cells grown in the logarithmic phase were plated in six-well plates at a density of 10^5 cells per well and incubated for 12 h. When the cellular confluence reached 80–90%, 1000 μl pipette tips were used to create wounds in the confluent cells. Then the cells were washed with PBS and incubated in a RPMI-1640 medium containing 10% FBS. Multiple photographs were then taken at 0 h, 12 h, 24 h, and 48 h at the same spots.

### Clone formation assay

Treated cells were incubated onto six-well plates (1000 cells per well) or twelve-well plates (500 cells per well) containing 1640 medium with 10% FBS and cultured for 2 weeks. Subsequently, colonies were fixed with paraformaldehyde and stained with crystal violet. Finally, the number of colonies was counted utilizing the software image J. This experiment was repeated at least three times.

### CCK-8 assay

Treated cells were cultivated on 96-well plates, with 2000 or 3000 cells per well and cultured for 24 h, 48 h, and 72 h. Then, 10 μl CCK-8 (APEXBIO, America) was added to each well with 100 μl medium at 37 °C for 2–4 h. Finally, Cell viability was determined by measuring the optical density at 450 nm.

### Cell cycle analysis

Treated cells were seeded in 6-well plates (1 × 10^5^ cells per well) with three replicates. After 48 h, cells were collected and washed twice with PBS and fixed with ice-cold 75% ethanol overnight. Then, the cells were washed with PBS twice and treated with RNase A for 30 min at 37 °C. Subsequently, the cells were then stained with PI and analyzed by flow cytometry. Finally, the percentage of cells in different cell cycle phases was analyzed with FlowJo software.

### Immunofluorescence

Cells with different treatments were respectively grown on three glass coverslips (15 mm diameter) and fixed in 4% paraformaldehyde (PFA) for 10 min at room temperature. The coverslips were washed in PBS three times for 5 min. Then the cells were permeabilized with 0.5% TritonX-100 for 5 min and briefly blocked with 1% bovine serum albumin (BSA) solution diluted by PBS for 10–15 min. Subsequently, the cells were incubated with ciliary marker antibodies against acetylated tubulin (1:500 dilution, Sigma-Aldrich) in PBS at 4 °C overnight. After incubating with TRITC-labeled anti-mouse secondary antibody (1:200 dilution, Southern Biotech) for 2 h, the nuclei were stained with 4′,6-diamino-2-phenylindole (DAPI, Solarbio). PC were observed in randomly selected 800× microscopic fields (2–5 fields in each glass coverslip).

### ELISA

The expression levels of STIL and AURKA in the blood of patients were determined by the enzyme-linked immunosorbent assay (ELISA) kits (Abbkine, China) according to the manual. The optical density (OD) values were detected by a microplate reader (spectral max 340, Molecular Devices, Sunnyvale, CA, USA) at 450 nm. The concentration of the samples was calculated to draw the standard according to the standard curve.

### RNA-sequencing (RNA-seq)

Total RNAs were isolated from stable STIL-knockdown EJ cells and their corresponding controls. Then the RNA-seq was conducted by the Beijing Genomics Institute (BGI) genomics utilizing the BGISEQ-500 instrument model. A propriety Sub-pixel Registration algorithm developed by MGI was used for the base call. This project uses the filtering software SOAP nuke developed by BGI independently for filtering. Bowtie2 was used to map the clean reads to the reference gene sequence (transcriptome), and then RSEM was used to calculate the gene expression level of each sample. Then the gene read count value was normalized and transformed to a CPM unit by utilizing the edgR packages, and then the data were conducted for subsequent differential expression analysis. Due to the small number of duplicate samples, genes with adjusted P-value < 0.01 were regarded as differential expression genes (DEGs) in this project. The full RNA-seq data set is available online http://www.ncbi.nlm.nih.gov/geo/ under the data series accession number GSE211563.

### Co-immunoprecipitation

The ThermoScientific Pierce co-IP kit was used to perform the Co-immunoprecipitation (Co-IP) following the manufacturer’s protocol. EJ cells were collected and added with precooled ice-cold IP Lysis/Wash Buffer with 1% PMSF and then placed on ice for 5 min. AminoLink Plus Coupling Resin was washed twice by adding 200 μl of 1× Coupling Buffer. Then, 20 μg of anti-STIL antibody (rabbit, Invitrogen), anti-AURKA antibody (rabbit, Invitrogen), and Mouse IgG antibody (B900620, proteintech) were immobilized using AminoLink Plus coupling resin for 1.5–2 h, respectively. After that, the lysate and antibody mixture slowly oscillated at 4 °C overnight. After elution and sample preparation, samples were analyzed by Western blotting.

### Xenograft model in nude mice

The xenograft tumour-growth assay was established by the subcutaneously of axilla injection of 2 * 10^6^ transfected EJ cells into Balb/c nude mice (4–6 weeks old, 5 mice/group). The tumour growth was identified by measuring tumour length (L) and width (W) each week, and the tumour volume was estimated using the formula: tumour volume = 0.52 × (L × W^2^). Four weeks later, the mice were given an intraperitoneal injection of the substrate luciferin (Promega, Fitchburg, WI, USA) at 100 mg/kg body weight. Then the mice were anaesthetized with isoflurane, and an IVIS^®^ Lumina II Imaging System (Caliper Life Sciences, Hopkinton, MA) was used to image the luciferase bio-luminance. Subsequently, the mice were euthanized, and their tumours were isolated and used to conduct western blot analysis. The equation of weight ratio is the weight of the tumour/weight of a mouse.

### Statistical analyses

Unpaired t-test analysis was utilized to compare the differences between the two groups. The log-rank test was utilized to estimate the prognosis of patients. The chi-square test was used to determine the difference between clinical information and different expression groups. Meanwhile, the Pearson correlation coefficient was a common method to assess the correlation between the two groups. The difference was considered statistically significant for all analyses when a P-value < 0.05 (Additional file 2).

## Supplementary Information

**Additional file 1: Figure S1.** PC is deficient in human bladder cancer cells, and PC assembly is enhanced under serum-starved conditions. (A) Normal bladder epithelium cells (SV-HUC-1) and bladder cancer cells (BIU87, EJ, T24, and 5637) were serum-starved for 48 h, then fixed and stained with antibodies against ace-tubulin (red) and DAPI (blue) for immunofluorescence analysis. The scale bar indicates 10 μm. (B) The percentage of ciliated cells in SV-HUC-1, BIU87, EJ, T24, and 5637 cells. (C) Control, 24 h serum-starved, 48 h serum-starved, and serum re-stimulation after 48 h serum-starved EJ cells were stained with antibodies against ace-tubulin (red) and DAPI (blue) for immunofluorescence analysis. (D) The percentage of ciliated cells in Control, 24 h serum-starved, 48 h serum-starved, and serum re-stimulation after 48 h serum-starved EJ cells. (E) The expression levels of acetylated tubulin and ARL13B and quantification of Control, 24 h serum-starved, 48 h serum-starved, and serum re-stimulation after 48 h serum-starved EJ cells (n = 3, mean ± SEM). Unpaired t-test analysis was utilized to compare the differences between the two groups. *P-value < 0.05, **P-value < 0.01, ***P-value < 0.001, ****P-value < 0.0001. **Figure S2.** PC deficiency could promote the cell cycle and proliferation of BLCA cells. (A-C) SV-HUC-1 and EJ cells transfected with siNC and siIFT88 were serum-starved for 48 h, then fixed and stained antibodies against ace-tubulin (red) and DAPI (blue) for immunofluorescence analysis. The percentage of ciliated cells in different groups was quantification. (D) Western blot analysis for the protein expression (IFT88 and acetylated tubulin) in the control and IFT88-knockdown cells. The percentage of protein levels was quantification. (E) Cell cycle analysis of IFT88-knockdown and control BLCA cells. (F) The viability of IFT88-knockdown and control BLCA cells was measured by CCK8 assay. (G, H) Western blot analysis for the protein expression (including IFT88, acetylated tubulin, ARL13B, SHH, SMO, and GLI1) in IFT88-knockdown and control BLCA cells. The percentage of protein levels was quantification. Unpaired t-test analysis was utilized to compare the differences between the two groups. *P-value < 0.05, **P-value < 0.01, ***P-value < 0.001, ****P-value < 0.0001. **Figure S3.** STIL regulates migration, proliferation, growth, and cycle in T24 cells. (A, B) The Kaplan–Meier survival curves were depicted to show the progression-free survival (PFS) and recurrence-free survival (RFS) in the high/low STIL expression group. (C, D) The migration ability for STIL knockdown/over-expression T24 cells compared to their corresponding control group by the wound healing assays. (E, F) The viability of STIL knockdown/over-expression T24 cells compared to their corresponding control group was measured by CCK8 assay. (G-I) The cell growth for STIL knockdown/over-expression T24 cells compared to their corresponding control group by colony formation assays. The number of colonies was counted utilizing the software image J. (J, K) Cell cycle analysis of STIL knockdown/over-expression T24 cells compared to their corresponding control group. Unpaired t-test analysis was utilized to compare the differences between the two groups. *P-value < 0.05, **P-value < 0.01, ***P-value < 0.001, ****P-value < 0.0001. **Figure S4.** STIL locates at the centrosome and regulates PC formation. (A) The EJ cell was serum-starved for 48 h, then fixed and stained antibodies against γ-tubulin (red), STIL (green), and DAPI (blue) for immunofluorescence analysis. (B) The expression levels of STIL and quantification of Control, 24 h serum-starved, 48 h serum-starved, and serum re-stimulation after 48 h serum-starved EJ cells (n = 3, mean ± SEM). Unpaired t-test analysis was utilized to compare the differences between the two groups. (C) No serum-starved and 48 h serum-starved EJ cells were stained antibodies against acetylated tubulin (red), STIL (green), and DAPI (blue) for immunofluorescence analysis. (D-E) The STIL knockdown or overexpression and their corresponding control T24 cells were serum-starved for 48 h, then fixed and stained antibodies against ace-tubulin (red) and DAPI (blue) for immunofluorescence analysis. The percentage of ciliated cells were compared between the control and treatment group. (F) Western blot analysis for the protein expression (including STIL, IFT88, acetylated tubulin, ARL13B, SMO, and GLI1) in STIL-knockdown and control T24 cells. The percentage of protein levels was quantification. (G) Western blot analysis for the protein expression (including STIL, IFT88, acetylated tubulin, ARL13B, SHH, SMO, and GLI1) in STIL-overexpression and control T24 cells. The percentage of protein levels was quantification. (H) Control and STIL over-expressed T24 cells were fixed and stained with antibodies against GLI1 (red) for immunofluorescence analysis. The scale bar indicates 10 μm. (I) siNC and siSTIL T24 cells transfected with siIFT88 or not, respectively, were serum-starved for 48 h, then fixed and stained with antibodies against ace-tubulin (red) and DAPI (blue) for immunofluorescence analysis. The scale bar indicates 10 μm. The percentage of ciliated cells was quantification. (J and K) The protein expression levels (including STIL, acetylated tubulin, ARL13B, IFT88, SHH, and GLI1) in siNC and siSTIL T24 cells transfected with siIFT88 or not were respectively detected by western blot analysis. All the percentages of protein levels were quantification. Unpaired t-test analysis was utilized to compare the differences between the two groups. *P-value < 0.05, **P-value < 0.01, ***P-value < 0.001, ****P-value < 0.0001. **Figure S5.** STIL regulated AURKA in T24 cells. (A, B) The Kaplan–Meier survival curves were depicted to show the progression-free survival (PFS) and recurrence-free survival (RFS) in high/low AURKA expression. (C, D) The western blot analysis for AURKA protein expression in STIL depletion/overexpression T24 cells and their corresponding normal control. (E, F) The western blot analysis for the protein expression (STIL, AURKA, acetylated tubulin, ARL13B, and IFT88) in AURKA depletion/overexpression T24 cells and their corresponding normal control. (G) The protein expression levels (STIL, AURKA, acetylated tubulin, ARL13B, IFT88, SHH, GLI1, and SMO) in siNC and siSTIL T24 cells transfected with siNC or siAURKA respectively were detected by western blot analysis. (H) The protein expression levels (STIL, AURKA, acetylated tubulin, ARL13B, IFT88, SHH, GLI1, and SMO) in control and STIL overexpressed T24 cells transfected with siNC or siAURKA respectively were detected by western blot analysis. All the percentages of protein levels were quantification. Unpaired t-test analysis was utilized to compare the differences between the two groups. *P-value < 0.05, **P-value < 0.01, ***P-value < 0.001, ****P-value < 0.0001. **Figure S6.** STIL influences cell migration, proliferation, growth, and PC formation by regulating AURKA. (A, B) siNC and siSTIL T24 cells transfected with siNC or siAURKA, respectively, were serum-starved for 48 h, then fixed and stained with antibodies against ace-tubulin (red) and DAPI (blue) for immunofluorescence analysis. The scale bar indicates 10 μm. The percentage of ciliated cells was quantification. (C, D) Control and STIL overexpressed T24 cells transfected with siNC or siAURKA respectively were serum-starved for 48 h, then fixed and stained with antibodies against ace-tubulin (red) and DAPI (blue) for immunofluorescence analysis. The scale bar indicates 10 μm. The percentage of ciliated cells was quantification. (E) The migration of control and STIL overexpressed T24 cells transfected with siNC or siAURKA were analyzed by wound-healing assay. (F, G) The growth and viability of control and STIL overexpressed T24 cells transfected with siNC or siAURKA were analyzed by CCK8 and colony formation assay. (H, I) The western blot analysis for protein expression (STIL and AURKA) in vector and STIL overexpressed T24 cells treated with different concentrations of cycloheximide (CHX). (J, K) The western blot analysis for protein expression (STIL and AURKA) in siNC and siSTIL T24 cells treated with different concentrations of cycloheximide (CHX). All the percentages of protein levels were quantification. Unpaired t-test analysis was utilized to compare the differences between the two groups. *P-value < 0.05, **P-value < 0.01, ***P-value < 0.001, ****P-value < 0.0001.

**Additional file 2.** Additional file for peer-review.

**Additional file 3: Table S1.** Summary of information on siRNA sequences.

## Data Availability

All relevant data are within the paper and its Supporting Information.
