# Supplementary figures and images for: STIL/AURKA axis promotes cell proliferation by influencing primary cilia formation in bladder cancer
Source: J Transl Med. 2023 Apr 26;21:281. doi: 10.1186/s12967-023-04118-2 (PMC10131372; doi:10.1186/s12967-023-04118-2)

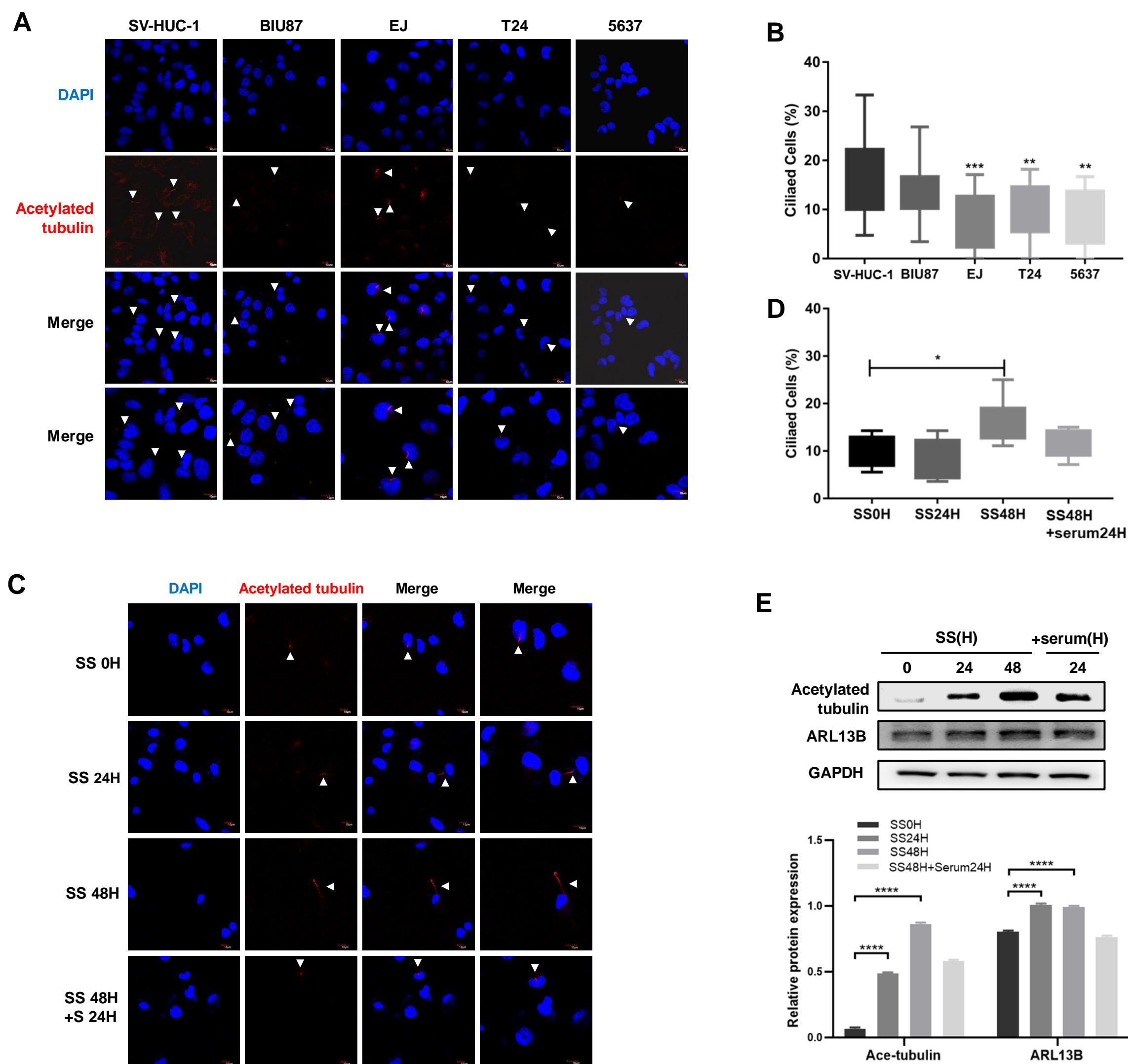

Figure S1

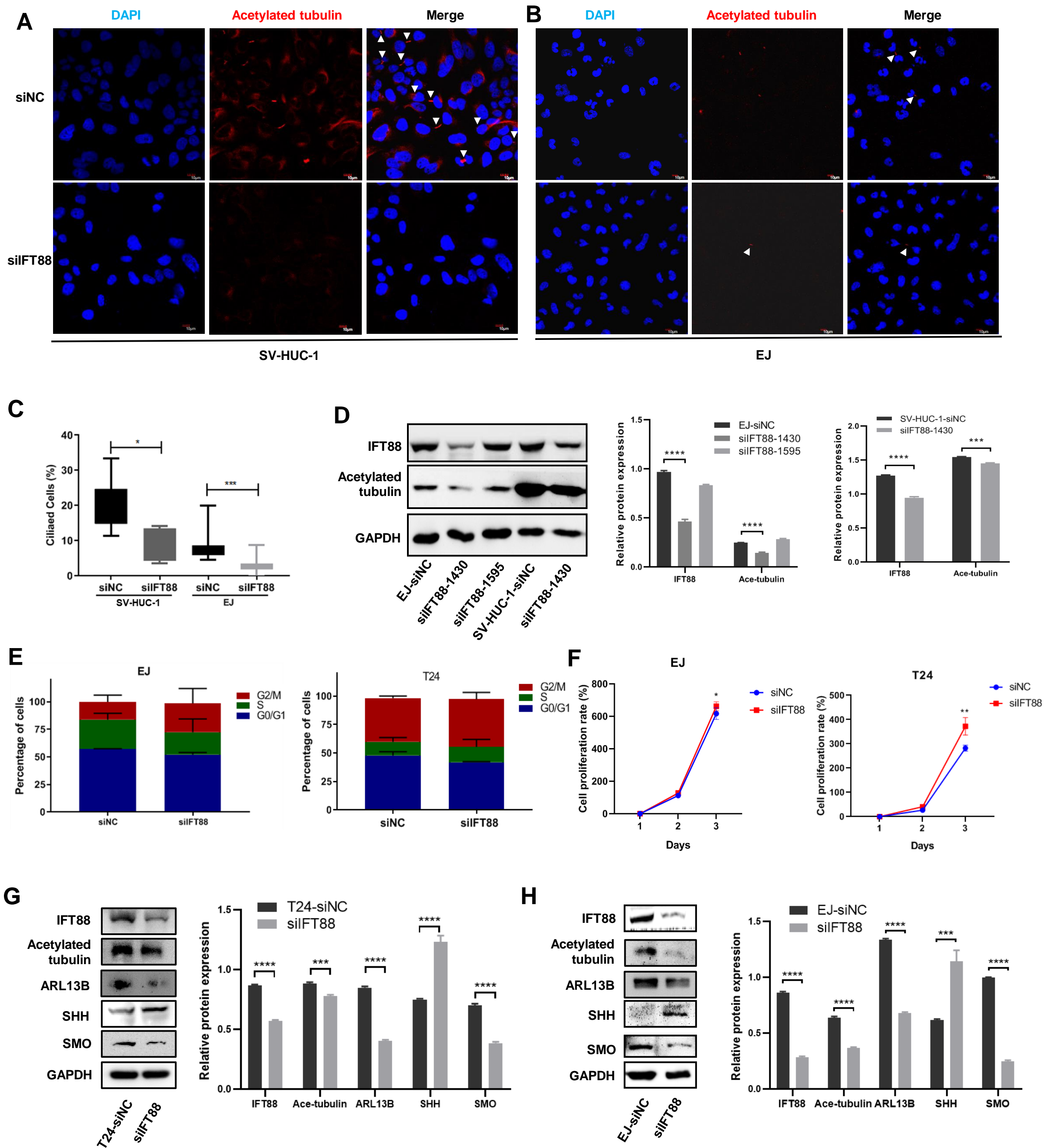

**Figure S2**

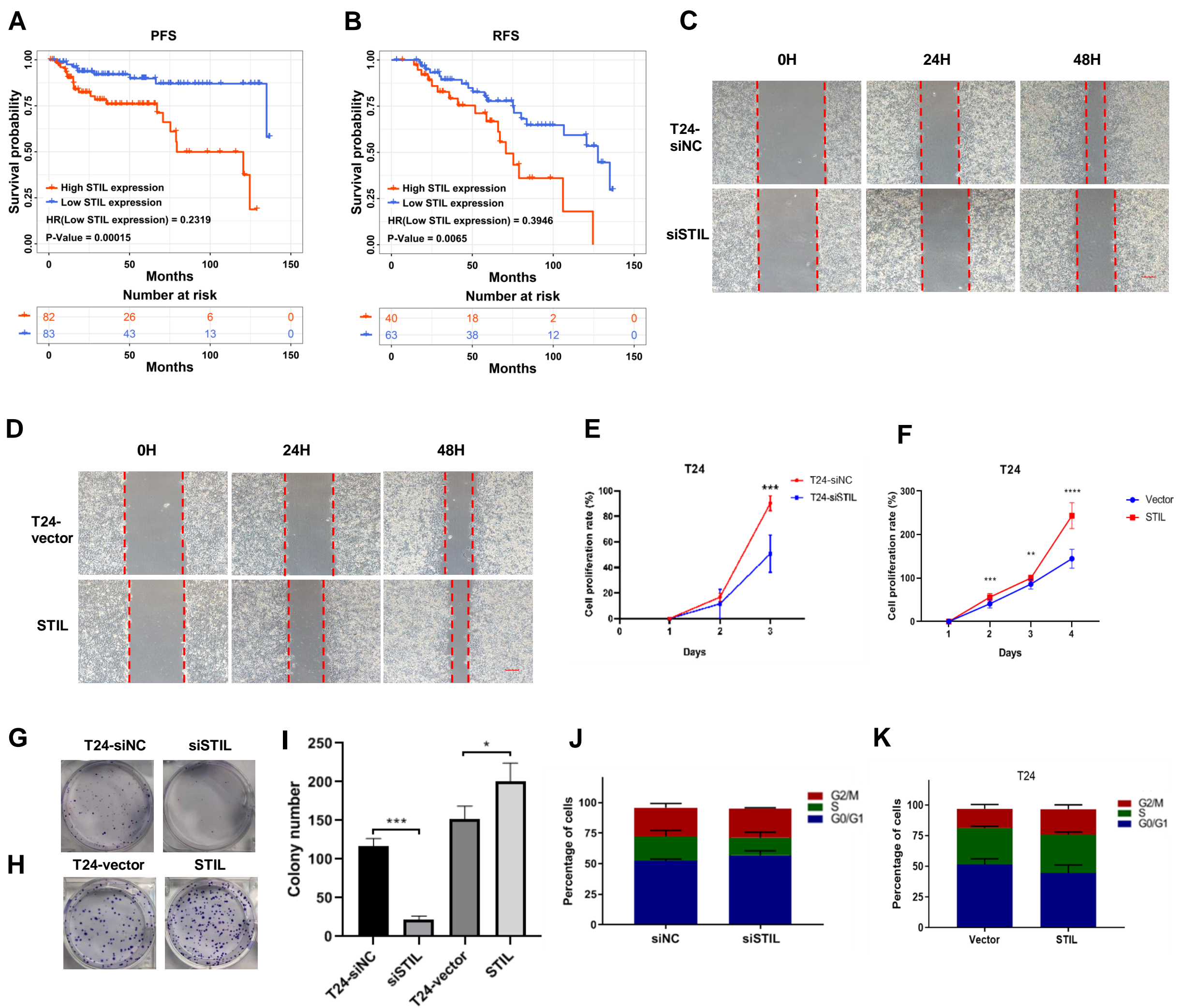

Figure S3

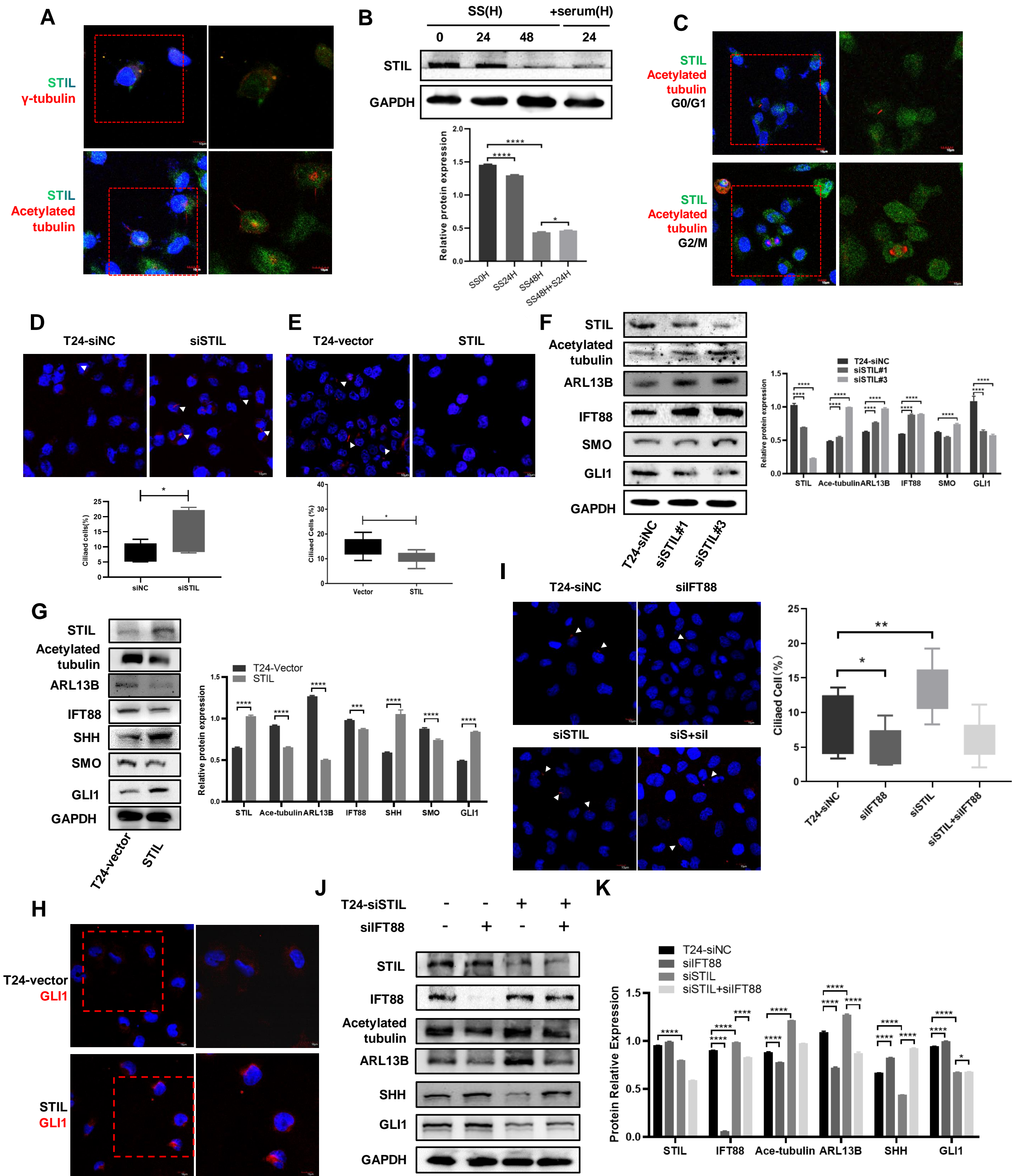

**Figure S4**

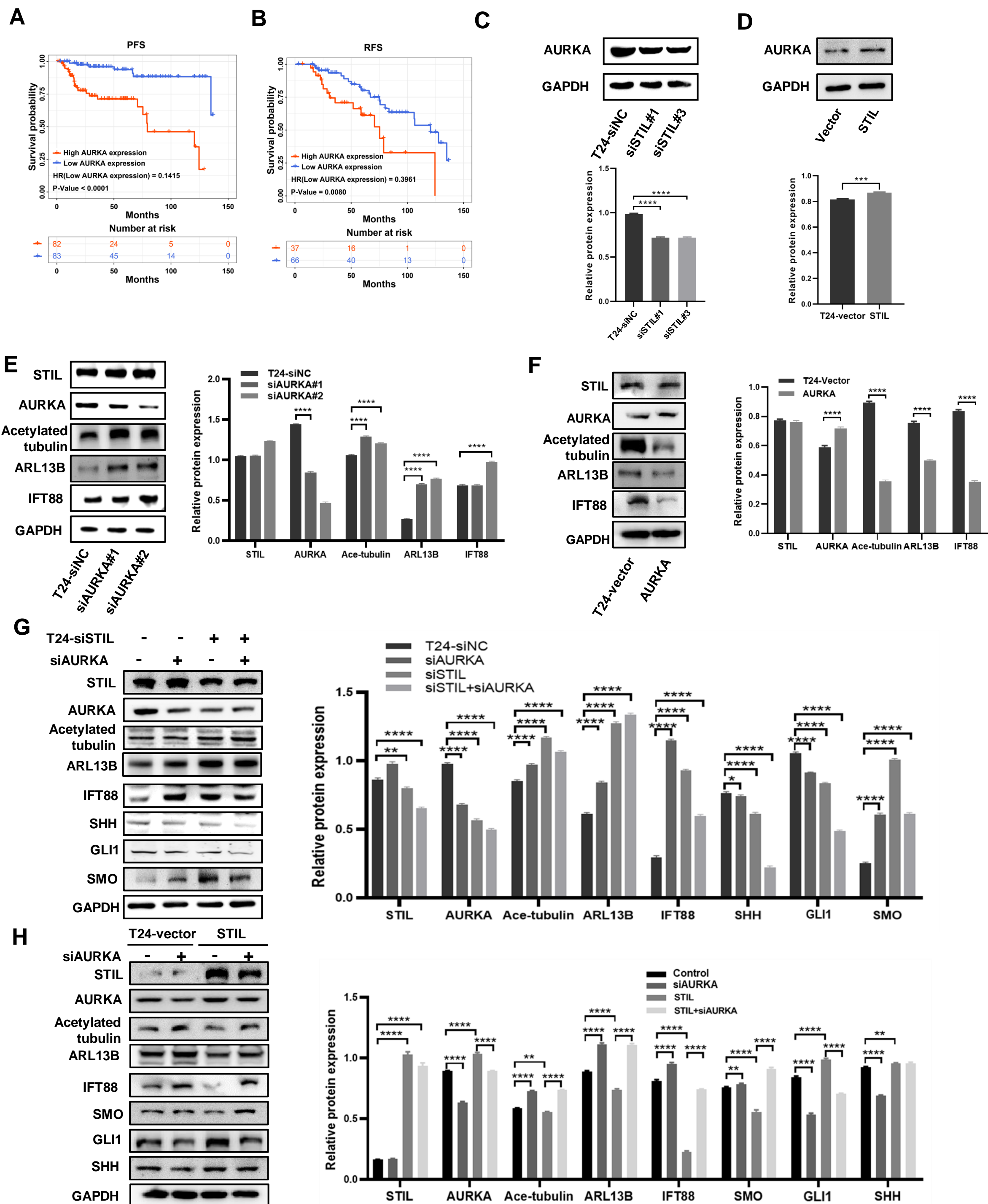

**Figure S5**

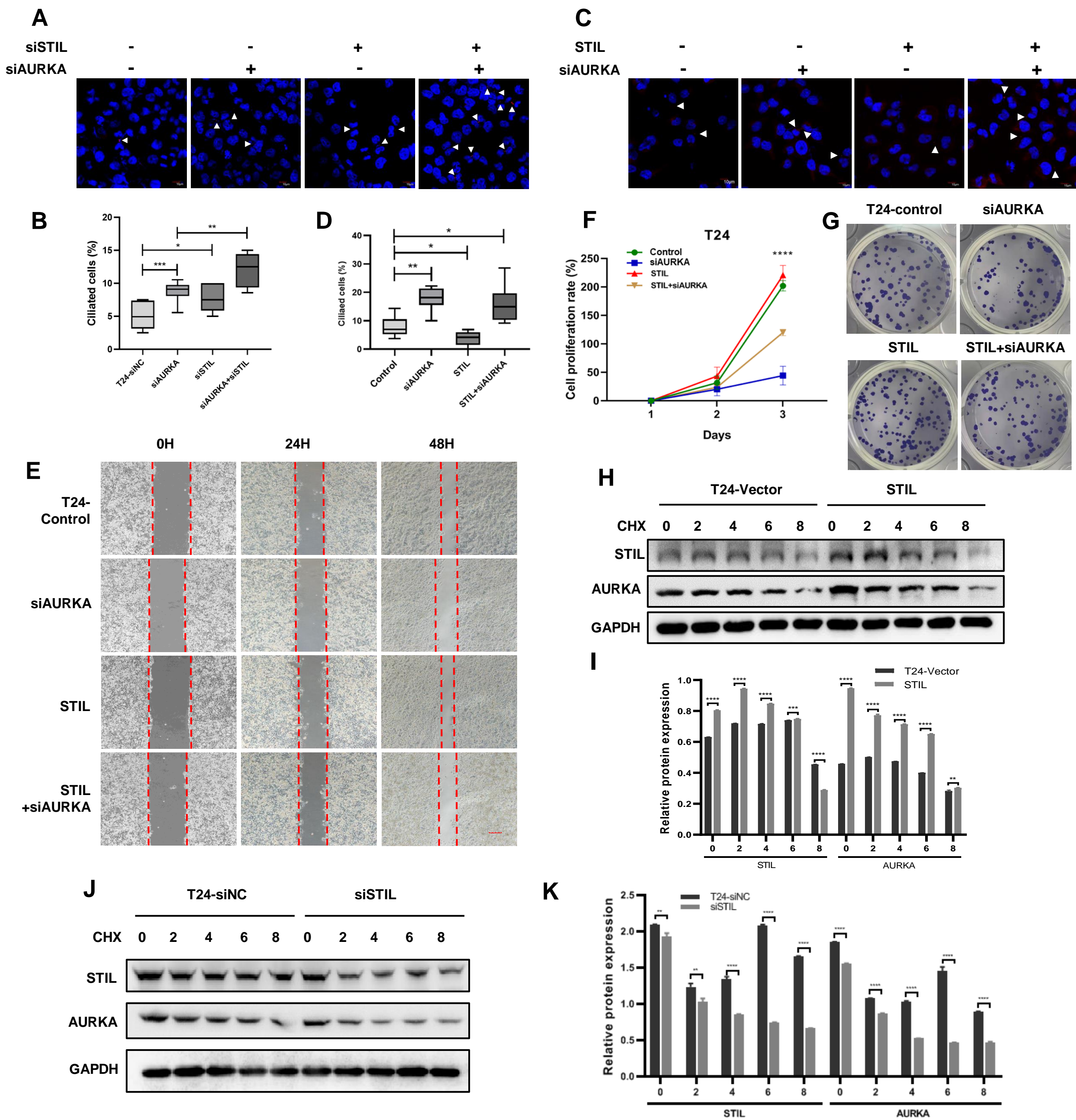

Figure S6

Supplement: Supplementary file 1 — Additional file 1: Figure S1. PC is deficient in human bladder cancer cells, and PC assembly is enhanced under serum-starved conditions. (A) Normal bladder epithelium cells (SV-HUC-1) and bladder cancer cells (BIU87, EJ, T24, and 5637) were serum-starved for 48 h, then fixed and stained with antibodies against ace-tubulin (red) and DAPI (blue) for immunofluorescence analysis. The scale bar indicates 10 μm. (B) The percentage of ciliated cells in SV-HUC-1, BIU87, EJ, T24, and 5637 cells. (C) Control, 24 h serum-starved, 48 h serum-starved, and serum re-stimulation after 48 h serum-starved EJ cells were stained with antibodies against ace-tubulin (red) and DAPI (blue) for immunofluorescence analysis. (D) The percentage of ciliated cells in Control, 24 h serum-starved, 48 h serum-starved, and serum re-stimulation after 48 h serum-starved EJ cells. (E) The expression levels of acetylated tubulin and ARL13B and quantification of Control, 24 h serum-starved, 48 h serum-starved, and serum re-stimulation after 48 h serum-starved EJ cells (n = 3, mean ± SEM). Unpaired t-test analysis was utilized to compare the differences between the two groups. *P-value < 0.05, **P-value < 0.01, ***P-value < 0.001, ****P-value < 0.0001. Figure S2. PC deficiency could promote the cell cycle and proliferation of BLCA cells. (A-C) SV-HUC-1 and EJ cells transfected with siNC and siIFT88 were serum-starved for 48 h, then fixed and stained antibodies against ace-tubulin (red) and DAPI (blue) for immunofluorescence analysis. The percentage of ciliated cells in different groups was quantification. (D) Western blot analysis for the protein expression (IFT88 and acetylated tubulin) in the control and IFT88-knockdown cells. The percentage of protein levels was quantification. (E) Cell cycle analysis of IFT88-knockdown and control BLCA cells. (F) The viability of IFT88-knockdown and control BLCA cells was measured by CCK8 assay. (G, H) Western blot analysis for the protein expression (including IFT88, [file 12967_2023_4118_MOESM1_ESM.pdf]
